# Machine learning-based forecasting of daily acute ischemic stroke admissions using weather data

**DOI:** 10.1038/s41746-025-01619-w

**Published:** 2025-04-25

**Authors:** Nandhini Santhanam, Hee E. Kim, David Rügamer, Andreas Bender, Stefan Muthers, Chang Gyu Cho, Angelika Alonso, Kristina Szabo, Franz-Simon Centner, Holger Wenz, Thomas Ganslandt, Michael Platten, Christoph Groden, Michael Neumaier, Fabian Siegel, Máté E. Maros

**Affiliations:** 1 Department of Biomedical Informatics at the Center for Preventive Medicine and Digital Health, Medical Faculty Mannheim, Heidelberg University, Mannheim, Germany; 2 Department of Statistics, LMU Munich, Munich, Germany; 3 Munich Center for Machine Learning (MCML), Munich, Germany; 4 Research Centre Human Biometeorology, German Weather Service (DWD), Freiburg, Germany; 5 Department of Neuroradiology, Medical Faculty Mannheim, Heidelberg University, Mannheim, Germany; 6 Clinic for Neurology, Medical Faculty Mannheim, Heidelberg University, Mannheim, Germany; 7 Department of Anesthesiology, Surgical Intensive Care Medicine and Pain Medicine, Medical Faculty Mannheim, University of Heidelberg, Mannheim, Germany; 8 Chair of Medical Informatics, Friedrich-Alexander-Universität Erlangen-Nürnberg, Erlangen, Germany; 9 Institute for Clinical Chemistry, Medical Faculty Mannheim, Heidelberg University, Mannheim, Germany

**Keywords:** Health care, Environmental health, Stroke

## Abstract

The climate crisis underscores the need for weather-based predictive analytics in healthcare, as weather factors contribute to ~11% of the global stroke burden. Therefore, we developed machine learning models using locoregional weather data to forecast daily acute ischemic stroke (AIS) admissions. An AIS cohort of 7914 patients admitted between 2015 and 2021 at the tertiary University Medical Center Mannheim, Germany, with a 600,000-population catchment area, was geospatially matched to German Weather Service data. Poisson regression, boosted generalized additive models, support vector machines, random forest, and extreme gradient boosting (XGB) were evaluated within a time-stratified nested cross-validation framework. XGB performed best (mean absolute error: 1.21 cases/day). Maximum air pressure was the top predictor, with temperature exhibiting a bimodal link. Cold and heat stressor days (*T*_min_lag3_ < −2 °C; *T*_perceived_ < −1.4 °C; *T*_min_lag7_ > 15 °C) and stormy conditions (wind gusts > 14 m/s) increased stroke admissions. This generalizable framework could aid real-time hospital planning, effective care and forecasting of various weather-related disease burdens.

## Introduction

The intensifying climate crisis poses a severe threat to ecosystems and human well-being, particularly to ageing populations^1–3^. Stroke is a major contributor to the global burden of cardiovascular disease, requiring prompt treatment for effectiveness^1^. However, current healthcare systems struggle to dynamically adapt to weather related fluctuations in demand^2,3^. This study leverages machine learning (ML) to develop predictive models using meteorological data to forecast acute ischemic stroke (AIS) admissions, aiming to enhance healthcare planning and accelerate responses to weather-related health incidents.

As acute ischemic stroke (AIS) accounts for the majority (60–70%) of all strokes and has a distinct etiology involving vessel thrombosis^1^, along with the availability of highly effective treatment options such as thrombolysis and mechanical thrombectomy^4^, our study explicitly focused on predicting this subcohort. In addition to individual risk factors, various weather conditions have been linked to stroke occurrences, including extremes of ambient temperature^5–9^, atmospheric pressure^10–12^, wind speed^13,14^, and ambient particulate matter with a diameter of <2.5 μm (PM_2.5_) pollution^15,16^. Some studies have found a positive association between higher temperatures due to heat stress^8,9^, higher air pressure^11,12^, and higher wind speed^13^ leading to an increase in stroke occurrences. In contrast, other studies have established a negative link between AIS admissions and cooler temperatures^5–7^, lower air pressure^10^, as well as lower wind speed^14^. While certain studies found no relevant association between weather conditions and the occurrence of stroke^8,17^. These inconsistencies may be attributed to the differing effects of temperature extremes, as temperature influences ischemic stroke risk through distinct mechanisms^18,19^. Heat stress has an immediate effect, accelerating dehydration, endothelial damage, and rheological changes that promote thrombosis, while cold stress, with a longer lag, induces vasoconstriction, elevated blood pressure, and vascular strain, with seemingly stronger overall effect regardless of the seasons^18–20^. Additionally, humidity can exacerbate these effects by further influencing dehydration and blood viscosity^19,21^.

Although ML models have been previously used to predict the number of admission counts for various diseases based on weather data, such as heat strokes, cerebrovascular, and overall emergency room visits^22,23^, none of these studies are concerned with ischemic stroke admissions. Furthermore, they were neither intended for forecasting nor did they fully exploit the extensive array of weather features and lagged parameters to develop an open-source comprehensive predictive framework.

Therefore, this study aimed to develop and benchmark ML-based predictive models for AIS admissions using geospatially matched locoregional weather parameters for clinically relevant daily time resolution. We employed a time-stratified 5 × 5-fold nested cross-validation setup over a seven-year period for a tertiary university clinic with a catchment area of 600,000 population. Our results underscore the potential of ML algorithms to forecast AIS admissions based on weather patterns, enabling improved resource allocation in the midst of climate change and providing a generalizable open-source framework applicable to various diseases.

## Results

### Study cohort

A single-center retrospective cohort of 7914 (4244 male, 53.6%) patients admitted with AIS between 2015 and 2021 at the UMC Mannheim, Germany, was retrieved from the local DIC. The average age of patients was 71 years (range: 7–98 years, SD = 14 years). The descriptive statistics for stroke admissions in the cohort and the distribution of the weather parameters such as temperature [°C], relative humidity [%], pressure [hPa], and windspeed [m/s] in the feature space (overall *n* = 133 variables) were summarized in Table 1.

**Table 1:** Summary table of the study cohort including the distributions of ischemic stroke admissions and weather parameters

Time frame	Variable	Median; LQ–UQ (min–max)
Overall	Total # of ischemic case admissions	3; 2–4 (0–10)
	Female	1; 1–2 (0–6)
	Male	2; 1–2 (0–7)
	Age < 70 years	1; 1–2 (0–6)
	Age 70 years	2; 1–3 (0–8)
Daily	Admissions on weekdays (Mo–Fr) | weekends (Sa–Su)	3; 2–4 (0–10) | 2; 1–3 (0–7)
	Admission time:	
	07:00–16:30	2; 1–2 (0–6)
	16:30–24:00	1; 1–1 (0–4)
	24:00–07:00	1; 1–2 (0–5)
	Admissions from the top 3 postal codes	1721, (29.2%) cases
	Admissions from the top 10 postal codes	4173, (47.1%) cases
Two-day	Total # of admissions	5; 4–7 (0–15)
Weekly	Total # of admissions	18; 15–21 (1–32)
Weather	Air temperature [°C]	11.3; 5.7-17.4 (-8.2–30.2)
	Relative humidity [%]	76.1; 64.2–85.6 (31.5–100)
	Air pressure [hPa]	1003.5; 994.7–1008.9 (912.7–1033.2)
	Wind speed [m/s]	2.7; 2.0–3.7 (0.9–18.1)

*#* number, *LQ* lower quartile, *UQ* upper quartile, *min.* minimum, *max.* maximum.

**Fig. 1:**
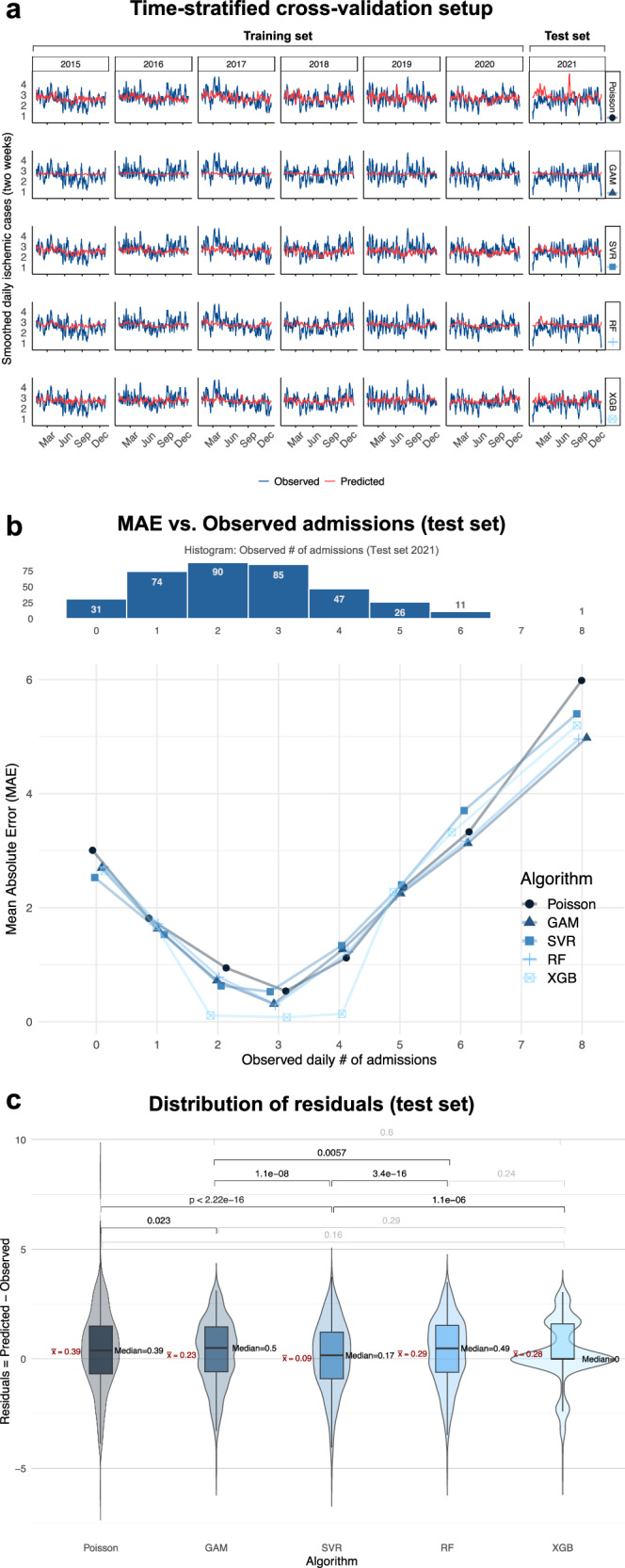
Setup for developing and benchmarking machine learning (ML) models to predict daily ischemic stroke admissions and their performance. **a** Six years (2015–2020; *n* = 2190 days) constituted the training set, wherein 5 × 5-fold, time-stratified, nested cross-validation was performed to optimize hyperparameters of the benchmarked ML models. The optimized models were then applied to the hold-out test set (2021; *n* = 365 days) in a regression setting. The investigated ML models (horizontal facet panels) included both well-established statistical models like Poisson regression (baseline) and boosted generalized additive models (GAM) as well as shallow ML algorithms such as support vector regression (SVR), random forest (RF) and extreme gradient boosting (XGB). For each year (vertical facet panels), the daily number of observed (blue lines) and ML-predicted (red lines) AIS cases were smoothed for a two-week period. **b** Combination plot showing the histogram (top) of the observed number of AIS admission in the test set (2021), alongside the mean absolute error (MAE) of the respective ML model (center, blue shades). XGB outperformed all other models achieving near-zero MAE within the 2–4 cases range. **c** Box- and violin plots of residuals (predicted-observed) with mean ($\bar{X}$) and median values, along with corresponding *p*-values (signif. in black) of pairwise Wilcoxon signed-rank tests (*N*_tests _= 10) after Holm correction. XGB showed the widest distribution around 0 ($\bar{X}$ = 0.28, median = 0). Although SVR had the lowest $\bar{X}$ = 0.09 (median = 0.17), it produced a broader range of predictions, resulting in significantly lower MAE compared to Poisson (median = $\bar{X}$ = 0.39, *p* = 2.2 × 10^−16^), GAM ($\bar{X}$ = 0.23, median = 0.5, *p* = 1.1 × 10^−8^), RF ($\bar{X}$ = 0.29, median = 0.49, *p* = 3.4 × 10^−6^) and XGB (*p* = 1.1 × 10^−6^). RF showed residuals similarly narrow to XGB (*p* = 0.24). Only XGB effectively learned the quantized prediction space of patient counts, while only Poisson predicted very rare days with >5 admissions (Supplementary Fig. 1c, d).

The autocorrelation function (ACF) and partial autocorrelation function (PACF) plots (Supplementary Fig. 1a, b) of daily AIS cases showed no significant autocorrelation, indicating that time series data could be considered sufficiently stationary. Therefore, classical statistical and shallow machine learning models were evaluated within the same time-stratified 5 × 5-fold nested cross-validation (CV) setup (Fig. 1a) with a training-validation set ranging from 2015 to 2020 (6 years, 2190 days) and 2021 (365 days) serving as the test set.

### Yearly and seasonal trends

**Fig. 2:**
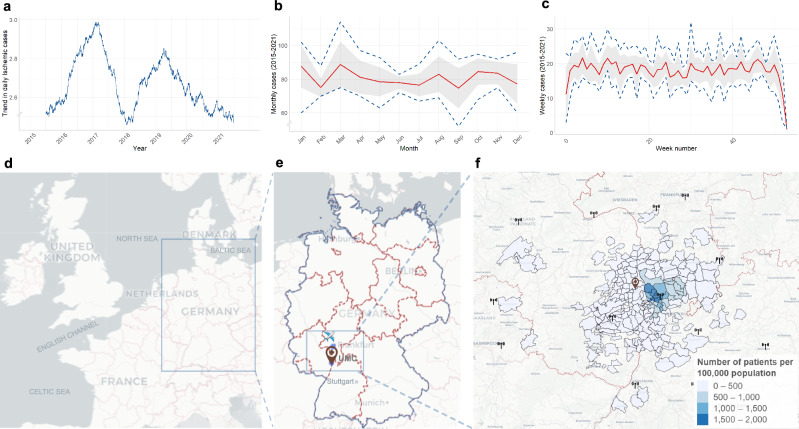
Combination figure of yearly, monthly, and weekly acute ischemic stroke admission (AIS) and their geospatial distribution. **a** Yearly trend analysis of AIS case counts showed a pronounced increase from 2015 to 2017, resembling hype cycles, potentially attributable to landmark clinical trials for the endovascular treatment of AIS^57^. In contrast, during the early COVID-19 pandemic (2020–2021), a clearly decreasing trend was observed. **b** Monthly AIS admissions (averaged over the 7-year study period; red line) indicated seasonal peaks in March, October, and November (95% CI in shaded gray) with min–max. ranges (dark blue dashed lines). **c** Weekly averages showed no apparent trends except for noticeable dips during the holiday season (50th-2nd weeks). **d** The University Medical Center Mannheim, Germany (UMC;  is located in the state of Baden–Wuerttemberg at (**e**) the corner of a German tri-state area (Rhineland Palatinate and Hesse; light blue bounding box). UMC is the primary tertiary care provider in Mannheim, the largest city in the region and the second largest in the state, with a population of 310,000 and a catchment area of over 600,000 people between Frankfurt () and Stuttgart. **f** Geospatial distribution highlighting the density of ischemic strokes per 100,000 population in the catchment area of UMC using the postal code-based distribution of patients’ home locations. The top three contributing areas were within an <11 km radius of the clinic and accounted for 29.2% of the total patient count, while 96.2% of all admissions arrived from a <50 km range. The selected weather stations are indicated with the icon ().

Yearly trends in AIS admissions displayed a significant increase in 2015 with subsequent declines in 2018 and 2020 (Fig. 2a). Analysis of aggregated monthly data over the 7-year period revealed pronounced seasonal variations with peak incidences occurring in March (mean = 88.85, 95% CI: 75.47–102.24, *p* = 3.46 $\times$10^−6^) and a decrease in September (mean = 74.71, 95% CI: 62.64–86.78, *p* = 5.21 $\times$10^−6^, Fig. 2b) followed by a secondary peak in October (mean = 84.57, 95% CI: 76.65–92.48, *p* = 2.06 $\times$10^−7^) and November (mean = 83.71, 95% CI: 77.18–90.24, *p* = 6.89 $\times$10^−6^). In contrast, if the case count was averaged over the week (weeks 1–52) over the 7-year period, no consistent pattern could be observed (Fig. 2c) other than noticeable dips during the holiday season (50th–2nd weeks).

### Spatial distributions

The UMC Mannheim is located in the state of Baden–Wuerttemberg (Fig. 2d, e) in the largest city of the metropolitan region Rhine–Neckar. Postal code-based geospatial analyses of patients’ home locations showed that the top three contributing areas were within a <11 km radius of the clinic and contributed to 29.2% of the total patient count. Over the span of seven years, patient counts from these regions ranged between 400 and 600 admissions/year (Fig. 2f). Similarly, when assessing the prevalence of the condition on a standardized rate per 100,000 population, these three postal codes consistently emerged as the top contributors. Overall ~96.2% of admissions came from within 50 km radius of the clinic supporting our locoregional approach and assumptions.

### Baseline statistical models

The baseline Poisson model (Supplementary Table 1) estimated the lag-5 mean cloud cover (OR = 0.97, 95% CI: 0.93–0.98, *p* = 0.0032) and lag-1 mean pressure (*P*_mean_lag1_; OR = 0.45, 95% CI: 0.24–0.81, *p* = 0.0076) as well as mean cloud cover (OR = 0.96, 95% CI: 0.94-0.99, *p* = 0.018) to be negatively correlated with AIS admissions. Conversely, lag-2 minimum temperature (*T*_min_lag2_; OR = 1.10, 95% CI: 1.01–1.20, *p* = 0.025) and maximum wind gust (*V*_gust_max_; OR = 1.02, 95% CI: 1.00–1.04, *p* = 0.017) were positively associated with increased daily case counts. Overall, the Poisson model had the largest prediction range (min–max: 0.99–8.56, median = 2.74, mean = 2.86, LQ–UQ: 2.36–3.19, IQR = 0.83) and highest absolute and residual errors (Fig. 1b, c), but it was the only model which predicted >5 admissions (Supplementary Fig. 1b, c).

The generalized additive models (GAM) identified maximum (*P*_max_) and mean pressures (*P*_mean_) as the two most influential variables with a reduction score with respect to root mean square error (RMSE) in percentages of 0.85 and 0.53, respectively (Supplementary Fig. 2a). Weekends (Saturday–Sundays) as calendar status indicators were the third most influential feature with a reduction score of 0.33 while minimal PT (PT_min_) was the fourth (0.13). GAM had the narrowest prediction distribution (min–max: 1.79–3.19, median = 2.71, mean = 2.70, LQ–UQ: 2.57–2.85, IQR = 0.28; Supplementary Fig. 1c) and a narrow range of residuals like RF (min–max: 1.66–4.36, median = 2.76, mean = 2.76, LQ–UQ: 2.6–2.91, IQR = 0.31; Fig. 1b, c).

### Machine learning models

Among the ML models tested, extreme gradient boosting (XGB) demonstrated the highest performance in predicting daily AIS cases, with the lowest MAE of 1.21 cases/day and RMSE of 1.49 cases/day in the test set. This represented a reduction of ~29% in MAE and a 44% decrease in RMSE compared to the baseline Poisson model (Table 2). Notably, similar performance was observed when the model was exclusively trained with weather variables only (Table 2).

**Table 2:** Overview of baseline statistical and ML model performance metrics on test set (2021)

		Weather features only			Weather and calendar features		
Model type	Model name	MAE (*N*_count_/day)	MAPE (%)	RMSE (*N*_count_/day)	MAE (*N*_count_/day)	MAPE (%)	RMSE (*N*_count_/day)
Baseline statistical	Poisson	1.72	58	2.73	1.69	57	2.68
	GAM	1.44	56	1.64	1.41	56	1.62
Machine learning (ML)	SVR	1.28	52	1.58	1.27	52	1.58
	RF	1.26	49	1.54	1.25	49	1.52
	XGB	1.25	48	1.52	1.21	47	1.49

*GAM* generalized additive model, *MAE* mean absolute error (*N*_count_/day), *MAPE* mean absolute percentage error (%), *RMSE* root mean square error (*N*_count_/day), *RF* random forest, *SVR* support vector regression using linear kernel, *XGB* extreme gradient boosting.

The XGB model effectively captured the variability of daily AIS case counts, especially for counts between 2 and 4 (near-zero MAE, Fig. 1b) in the test set (min–max: 1.67–4.05, median = 2.70, mean = 2.75, LQ–UQ: 2.56–3.00, IQR = 0.43). It had the lowest median residual error of 0 (Fig. 1c) and autonomously learned the discrete characteristic of the prediction space (Supplementary Fig. 1c). The distribution of SVR predictions (min–max: 1.34–4.17, median = 2.55, mean = 2.56, LQ–UQ: 2.22–2.86, IQR = 0.64) had the closest mean and median to the observed (min–max: 0–8, median = 2, mean = 2.47, LQ–UQ: 1–3, IQR = 2).

Notably, XGB encountered limitations when predicting days with either low (*n* = 0–1) or high (>5) AIS admission, which occurred on ~8.5% (31/365) and ~3.3% (12/365) of the days in the test set, respectively (Fig. 1b). This performance pattern mirrored the distribution of the training set from 2015–2020, where days with zero daily admissions occurred on 147 out of 2190 days (~6.7%), and days with >5 AIS cases occurred on 129 out of 2190 days (~5.9%), indicating no relevant data shift during the study period. Additionally, AIS admissions ranged between 2 and 5 cases/day on 67.4% of test set days and 69.4% of training set days.

### XGB-based variable importance of weather parameters

**Fig. 3:**
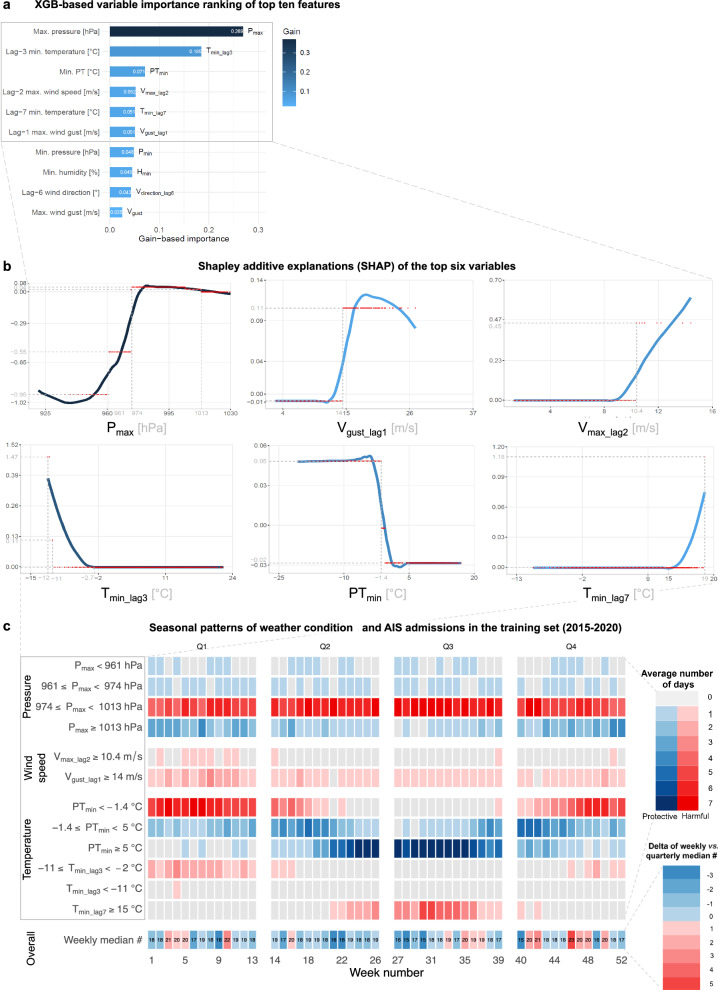
The composite figure of detailed analyses of the most important predictors of the best performing XGB model and their link to seasonal distribution of daily AIS admissions. **a** Horizontal bar chart of the top ten most relevant features using normalized gain-based variable importance ranking of the best XGB model. **b** Shapley additive explanations (SHAP) of the top six variables, including (upper-row) maximal air pressure (*P*_max_), lagged 1- and 2-days maximal wind speed (*V*_max_lag2_) and wind gust speeds (*V*_gust_lag1_); and (lower-row) minimal lagged 3-days temperature (*T*_min_lag3_), minimal perceived temperature (PT_min_), and 7-days minimum temperature (*T*_min_lag7_). These variables accounted for an overall sum of 0.84 gain-based importance out of the 133 investigated weather and calendar features. Inflection points on the subplots indicate (gray dashed lines) when the respective variable’s effect was associated with an increase or decrease in stroke counts. **c** Faceted heatmaps indicating the seasonal distributions of weather in the training data (2015–2020; *n* = 2190 days), thresholded using respective values from SHAP inflection points. The number of days that the respective condition has occurred was calculated by jointly aggregating at yearly and weekly levels (Supplementary Note 6. Aggregation methodology for the heatmap in Fig. 3, pp. 4). Protective (shades of blue) or harmful (red) median number of days were then color-coded based on the sign of the SHAP values. Additionally, the deltas of weekly stroke counts (aggregated over the seven years) were compared against the respective quarterly medians (lower right corner). *P*_max_ showed a sigmoid-like link as low pressures (*P*_max_ < 960 hPa) substantially decreased stroke admissions (SHAP = −0.95), while medium-high values (974–1013 hPa) were associated with an increased stroke incidence all year round (Q1–Q4). Cold stressor days (Q1, Q2, and Q4) and associated windy conditions (*V*max_lag_2_ $\ge$10.4 m/s and *V*_gust_max_lag1 _$\ge$14 m/s) substantially increased admissions (SHAPV_max_ = 0.11 and SHAP_Vgust_ = 0.45). Similarly, extended cold stressor periods during winter with *T*_min_lag3_ < −2 °C or PT_min_ < −1.4 °C were strongly linked to more strokes (SHAP up to 1.47). Conversely, PT_min_ in classical temperate ranges (−1.4 < PT_min_ < 20 °C) were slightly protective (SHAP = −0.03), although these effects could be outweighed (SHAP_Tmin_lag7_ = 1.18) during extended heat stress periods (_Tmin_lag7 _$\ge$15°C) of the summer.

*P*_max_ consistently emerged as the top variable for forecasting daily AIS case admissions across all deployed ML models. The XGB model distinguished itself by identifying lag-3 minimum temperature (*T*_min_lag3_) as the second most relevant variable (Fig. 3a); while SVR and RF models selected mean pressure (*P*_mean_) for this position (Supplementary Fig. 2b, c). Interestingly, PT_min_ emerged as the third-ranked predictor in both the XGB and RF models. The top 10 most important variables for XGB focused on temperature- and wind-speed-related features, while RF emphasized temperature- and vapor-pressure-based features. Besides weather variables, weekends also emerged among the top ten variables in the XGB model.

The SHAP-based analyses provided insights into how the top six meteorological parameters of XGB influenced the prediction of daily AIS admissions (Fig. 3b). *P*_max_ values were categorized into four ranges in post hoc analyses for a more detailed interpretation. During low-pressure conditions (<961 hPa), the SHAP was −0.95, indicating a reduction in AIS cases (Fig. 3b), which occurred on ~1.4% of the training days (31/2190) over a span of 24 weeks (Fig. 3c). The second range (961–974 hPa), also exhibited a protective effect, reducing ischemic cases by −0.55, despite being observed on only 2.5% of the days (56/2190). Conversely, the high-pressure (ranging 974–1013 hPa) conditions were positively associated with an increase in AIS case counts (SHAP = 0.04), occurring on 76% (1664/2190) of the training days without seasonal preference (Fig. 3c).

Temperature extremes played a dual role in AIS admissions, with both cold and hot stressor days positively associated with higher ischemic stroke counts. Especially, prolonged cold stressor periods with *T*_min_lag3_ < −11 °C were strongly linked (SHAP = 1.47) with a surge in AIS admissions (Fig. 3b). While such weather conditions were observed on only five days in the entire dataset, stroke admissions peaked between 7 and 9 cases exclusively on these days, specifically during January in the years 2017 and 2018 (Fig. 3c), however, AIS counts were generally lower on days surrounding these extreme cold periods. Additionally, PT_min_ < −1.4 °C underscored the positive impact of cold stress with an increased SHAP value of 0.05 (Fig. 3b). Such conditions were prevalent during the winter months (in the 1st and 4th quarters) and affected 36% (788/2190) of the training days (Fig. 3c). Similarly, prolonged hot stressors were also identified as triggers that increased ischemic stroke occurrences, with the 7-day lagged *T*_min_ (*T*_min_lag7_) ranked as the 5th most important variable by XGB (Fig. 3a). When *T*_min_lag7_ exceeded 15 °C (256/2190, 11.6%), the SHAP values steeply increased in a quasi-linear fashion from 0 to ~0.08 (Fig. 3b). If *T*_min_lag7_ reached 19 °C, the SHAP value escalated punctually to 1.18 (Fig. 3b). This phenomenon had a seasonal preference during the 3rd quarters, especially during the months of August and September in the years 2018–2020 (Fig. 3c).

Wind-related variables exhibited a distinct pattern indicating short-term effects on AIS admissions, particularly for lag-2 maximum wind speed (*V*_max_lag2_) and lag-1 maximum wind gust (*V*_gust_lag1_), which were associated with an increase in AIS cases (SHAP = 0.45 at *V*_max_lag2_ = 10.4 m/s and SAHP = 0.11 at *V*_gust_lag1_ = 14 m/s; Fig. 3b). These windy conditions occurred primarily during the first quarter, aligning with periods of cold stress and high-pressure stormy conditions (Fig. 3c).

## Discussion

We developed and benchmarked a set of well-established ML and statistical models to investigate the association between locoregional weather systems and the number of AIS admissions over a seven-year period to enable better planning of clinical resources. We found that shallow ML models were sufficient and outperformed baseline statistical models by 20–40% in terms of MAE and RMSE. XGB performed the best with an average MAE of 1.21- and RMSE of 1.49 cases/day, making it potentially useful for real-time forecasting. Regarding weather conditions, both cold and hot stressors days increased the number of daily stroke admissions, with prolonged colder conditions having a more prominent effect^18,19,21^. Additionally, high-pressure and stormy conditions tended to increase daily AIS admissions.

Our results highlight the potential application of ML models to forecast stroke occurrences based on weather- and seasonal patterns in real time for optimal clinical resource allocation and patient care. Real-time forecasting could be operationalized by integrating forward-facing predictions from established numerical weather prediction (NWP) models^24^ or novel deep learning-based weather forecasting systems^25–27^, which would provide weather feature inputs for the here proposed downstream ML models to predict upcoming clinical capacity requirements and optimize individual patient care or outcome in response to anticipated weather-driven admission trends^28–30^. By combining these methods, early warning systems might also be triggered in advance for vulnerable patients^31,32^, encouraging preventive measures to mitigate stroke risk and thereby reduce weather-driven admissions, or to facilitate secondary automated prevention programs^33^. Furthermore, this time-stratified, nested cross-validation setup provides a general framework that can be used for various diseases in multi-center applications, which can be easily adapted to different diseases and demographic profiles by modifying cohort selection criteria based on ICD codes and adjusting geographic regions through postal codes and corresponding weather data from regional weather services^34^. However, while these input features are generally available, enabling broad methodological transferability, anticipated challenges to generalization include heterogeneous availability, robustness, and quality of local weather data^35,36^, as well as substantial differences in healthcare infrastructure, such as data gaps and disparities in hospital accessibility between urban and rural areas^37^, especially in low- and middle-income countries^38^. These and other potentially unforeseen, context-specific factors may be more pronounced for certain diseases, thereby limiting ML model performance. Nevertheless, our open-source, ML-based precision medicine framework has the potential to optimize resource allocation and improve health equity by making EVT for stroke and other diseases more accessible and cost-effective^1,39,40^.

We observed a dual impact of temperature as both cold and hot stressors, especially over multiple days (>3 or 7 days), were associated with an increase in AIS counts, with a slight predominant effect of cold stress. This was consistent with findings from previous studies across diverse climatic zones globally^5–7,41–43^. A retrospective analysis of hospital data in the United States revealed a surge in stroke admissions during winter, accompanied by increased mortality rates^5^. Ambulance dispatches for ischemic stroke cases in Japan exhibited a similar seasonal pattern and were observed to be more common during lower temperatures^6^. Likewise, a retrospective study in China over a two-year period reported that 1.57% of ischemic strokes could be attributed to extremely cold temperatures, particularly in the 0–7 day lag period^7^. This aligns with our findings that prolonged cold stressors with *T*_min_ < −11 °C on three consecutive previous days substantially increased stroke incidence^18,19,21^.

Furthermore, both tree-based models (RF and XGB) identified PT_min_ as one of the top three predictors of daily AIS admissions, thereby emphasizing the importance of key human biometeorological features. PT_min_ showed a bimodal distribution of the estimated effects with higher weights given for cold days (PT_min_ < −1.4 °C). PT has certain advantages over other thermal indices, such as the wet-bulb globe temperature (WBGT) and the universal thermal climate index (UTCI)^44^, as it compares measured conditions to a reference subject (Climate-Michael) and environment while remaining robust against distortions from windy conditions and subzero temperatures^45^. It evaluates actual weather on an absolute and universally comparable scale in a season-independent and calibration-indifferent manner, making it particularly suitable for multi-centric applications across different climate regions (Supplementary Note 1. Klima-Michael model and perceived temperature, pp. 2)^44,45^.

Heat stress has also been observed to increase the incidence of AIS^8,9,46^. In a single-center retrospective study in Korea, Han et al. found that the seasonal AIS incidence in summer was significantly higher than in winter, and the mean temperature was positively associated with ischemic stroke with an RR of 1.006^8^. In accordance with this, the baseline Poisson model in our study identified a positive association between the minimum lag-2 temperature and daily stroke cases, with an increase of ~11% for every 1 °C increase. Ma et al. utilized a universal thermal climate index to quantify the weather conditions in Beijing^9^ and identified that the risk of suffering an AIS increased with heat stress, especially in the 45–65 years age group.

Multiple studies have shown that fluctuations in atmospheric pressure and temperature can promote arterial blood pressure instability and hemodynamic changes in the circulatory system^13,42^. The association between atmospheric pressure and the incidence of stroke has been studied in multiple retrospective studies^10–12^. Jimenez et al. reported that the drop in atmospheric pressure compared to the previous day can largely explain seasonal and daily variations of stroke incidence^11^. Qi et al. also discovered that mean, minimum, and maximum barometric pressures showed statistically significant positive associations with ischemic stroke occurrences, and the colder season tended to be the more risk-prone^12^. Our study also strongly supports these findings, as all ML models and the GAM identified pressure-related variables as the primary predictor of daily AIS admissions.

Coupled with high-pressure, stormy phenomena, maximum wind speed, and wind gusts on the previous 2 days, were linked to an increased number of AIS cases in our cohort. Similarly, in a small, localized study comprised of 409 stroke patients admitted during a two-year period (2006-2007) on an island in South Korea, wind speed and wind chill index were identified to have positive associations with AIS cases, which was more pronounced in spring and winter^13^. This cumulative effect of cold and stormy conditions within the same seasonal window was also observed in our study, which implied a compounded effect. It is important to note, however, that air pressure measurements by the DWD towers are referenced back to the respective sea level. For our West German region, it meant either the North Sea or the Atlantic Ocean.

Most studies analyzing the association between meteorological parameters and the onset of ischemic stroke have predominantly utilized classical statistical models, such as Poisson regression or its variations^12,15,41^. Only a few studies have employed ML-based models to develop a predictive framework using weather parameters, while these focused on conditions like heatstroke or general emergency room admissions^22^. Ogata et al. developed predictive models to forecast heatstroke admissions for a 3-year time period for sixteen cities in Japan using models such as GLM, GAM, and XGB^22^. It is noteworthy that the number of daily heatstroke admissions in their study substantially outranged (up to 400+ cases/d) the daily AIS admissions in our cohort (0–10 cases/d). The conventional GAM model exceeded the performance of other models, registering the lowest RMSE of 2.47 cases on their test set (2018), while XGB achieved an RMSE of 3.28. In contrast, XGB showed the best performance with an RMSE of 1.49 on the hold-out test set (2021) of our study for daily AIS admissions, despite predicting sparse values with lower variance, which is expected to be technically more challenging. XGB effectively handled the sparse case counts in our data (2–4 cases/day) and autonomously learned the discretely quantized nature of the prediction space, likely due to its ability to capture non-linear relationships between weather predictors and stroke admissions. Its boosting framework further mitigates the effects of correlated features and enhances model robustness in imbalanced datasets, while also providing theoretically more reliable feature importance measures^47–50^. In contrast, RF was probably limited by the correlated input features, and SVR, being a minimal margin predictor, optimized errors around the mean but failed to capture the full distribution. Interestingly, only the Poisson model predicted rare outlier days with >5 admissions, suggesting that model ensembling might be warranted^49^.

To interpret the effect of weather patterns, we used both permutation-based and SHAP feature importance methods. The former evaluates the decrease in model performance when a feature is permuted^47–49^, offering a global perspective linked to model error, while SHAP calculates magnitude-based feature attributions, providing local interpretability for individual predictions and suitability for non-linear relationships^29,51,52^. Together, these methods complement each other by addressing both global and local perspectives on feature importance.

Besides shallow ML algorithms, we also explored various deep learning architectures^30^, albeit non-systematically, including recurrent neural networks (RNN) and long short-term memory (LSTM) networks^29,30^, and complex forecasting frameworks like NeuralProphet by Meta. However, the decision to use shallow ML models was justified by the absence of higher-order autoregressive associations (ACF, PACF) within the data set and the principle of parsimony (Occam’s razor)^53^. This approach allowed us to opt for the less hardware-intensive CPU-bound modeling setup that does not require GPUs, thereby making our pipeline deployable for multi-centric applications (WE-STORM) within the network of the German Medical Informatics Initiative (MII)^34^.

This study has certain limitations, as it was a single-center retrospective analysis. However, the substantial cohort size of ~8000 patients with a catchment area of ~600,000 individuals supports the reliability of our results^6^. Additionally, we utilized a fine-grained temporospatial matching method to select weather variables from various DWD stations corresponding to patients’ home locations and admission hours. The assumption that patients were in reasonable vicinity of their home address had to be made for downstream analyses. Over 96.2% of the admitted patients’ homes were located within a 50 km radius, wherein variations of weather patterns are expected to be minimal, thereby supporting the feasibility of this approach^43^. However, the remaining 3.8% of the cohort could represent potential confounders, such as patients traveling, being on holiday, or visiting relatives and suffering the ictus there. Thus, the effect of lagged variables might differ, but short-term local weather parameters such as *P*_max_, *P*_min_, PT_min_ would likely remain dominant^19–21^. It is noteworthy that we did not apply additional feature selection methods, but utilized the internal variable selection provided by the respective ML model during training and validation^22^. Despite the established association of air pollutants with cerebral and respiratory diseases in previous studies^15,16^, we could not include this data in our analyses due to the very low density of monitoring stations in the area. Based on findings of the global, regional, and national burden of stroke and its risk factors study by Feigin et al., non-optimal temperatures contribute ~6.6% (95% CI: 4.5–9.1) to the global burden of stroke in disability-adjusted life-years (DALYs), with low ambient temperatures (5.8%, 95% CI: 4.4–7.5)—similar to our findings—having a more pronounced effect compared to high ambient temperatures (0.8%, 95% CI: 0.1–1.6), and up to 10.9–11.3% (as combined upper 95% CI limit) in middle- to high-income countries for all stroke types^1^. Regardless, we found that ML models can predict the number of daily admissions with an acceptable MAE and RMSE of <1.5 cases/day, particularly for the most common band of daily ischemic cases between 2 and 5, covering ~70% of the investigated timeframe.

In conclusion, using a detailed temporal and geospatial matching technique, this study systematically compared baseline statistical and ML models to forecast the number of acute ischemic stroke admissions based on weather patterns. ML models outperformed classical statistical models, demonstrating their potential for real-time healthcare resource allocation. The best-performing model (XGB) identified atmospheric pressure, lagged temperature, PT_min_, and wind speed as the most important predictors of stroke occurrence. Our results further emphasize the dual role of temperature for both hot and cold stressor days and the crucial effect of prolonged stormy conditions. We developed a generalizable framework that can be applied to various diseases and easily deployed as multi-centric applications in data integration centers nationally and around the world to determine the impact of locoregional weather conditions and seasonal variations.

## Methods

### Patient selection

All patients admitted with suspected acute ischemic stroke between 2015-01-01 and 2021-12-31 at the University Medical Center (UMC) Mannheim, Germany, were retrieved from the local data integration center (DIC) using the core data set of the Medical Informatics Initiative (MII), which was based on standardized Health Level Seven International Fast Health Interoperability Resources (HL7 FHIR) specifications^54^. Patients were identified using the International Classification of Diseases, Tenth Revision, German Modification (ICD-10-GM) codes: I63.0-9. Besides hospital diagnoses, general demographic information such as age, sex, admission date, and patients’ home address (postal codes) was also extracted (Supplementary Note 2. Patient matching, pp. 2).

### Weather data

Weather data were retrieved from the open data server of the German Weather Service (DWD) using the *rdwd* package. It comprised 440 weather stations covering Germany. The stations measured various parameters, such as air temperature, relative humidity, pressure, wind speed, wind direction, sunshine duration, precipitation, and cloud cover, with different parameters being sampled at different temporal resolutions between 2015 and 2021. Because not all stations had all measurements, weather parameters with hourly and daily resolutions were collected from stations with full parameter coverage and based on the patients’ home locations (135/440, 30.7%) to optimize the balance between sufficiently detailed temporal resolution and data processing requirements (Supplementary Fig. 3). Weather features averaged over multiple days were represented as lagged variables, such as a 3-day average indicated by lag-3 or a 7-day average indicated by lag-7. Additionally, we calculated well-established human biometeorological parameters such as perceived temperature (PT), which is derived from the Climate–Michael model (Supplementary Note 1. Klima–Michael model and perceived temperature, pp. 2)^44^.

### Geospatial matching

We assumed that patients were either at home or near their homes when the ictus occurred. To counteract this potential dependency, we performed a geospatial matching by assigning each patient to two weather stations, one closest to their home location and one closest to the clinic’s (UMC) location. This approach allowed us to identify three different patient types (Supplementary Fig. 3). Weather features were extracted from these respective stations for the admission date and time, and the previous seven days (lag-1 to lag-7) and were transformed into daily, two-day, weekly, and monthly resolutions (Supplementary Table 2). To account for potential differences between weather data from the home and clinic stations, measurements from both stations were averaged to create a single predictor. As the stations were geographically close (0–25 km apart), weather variability was minimal, ensuring that the averaged features accurately represented overall conditions in the area. To adjust for seasonal- and long-term trends, we have also incorporated calendar-based variables such as weekdays or -ends, year, week number, and holiday indicators based on public and school holidays in the state of Baden-Wuerttemberg as additional features in the feature space.

### Machine learning setup

The entire ML workflow was implemented using the *caret* package in the open-source R language (v.4.1.1, R Core Team, Vienna, Austria). All ML and statistical analyses were reported in accordance with the recently updated guidance for reporting clinical prediction models that use regression or machine learning methods (Transparent Reporting of a multivariable prediction model for Individual Prognosis Or Diagnosis, TRIPOD + AI statement)^55^. We investigated well-established shallow ML algorithms, including support vector regressors (SVR) using linear kernel (*e1071* package) and tree-based models including random forest (RF; *randomForest* package) and extreme gradient boosting (XGB; *xgboost* package) to predict the number of ischemic cases for the respective time resolution in a regression setting. Both ML- and statistical models were comprehensively evaluated within the same time-stratified 5 × 5-fold nested cross-validation (CV) setup (Supplementary Note 3. Time-stratified nested cross-validation setup, pp. 3) with a training-validation set ranging from 2015 to 2020 and 2021 serving as the test set. (Fig. 1a). The root mean square error (RMSE) was used as a loss function. The respective hyperparameters of the investigated ML algorithms and GAM were tuned in the training-validation set with an additional nested CV using either the built-in tuning option of the model function (*gamboost*) or the framework (*tuneGrid*) of the caret^47^ package with a dedicated tuning grid for each ML algorithm (Supplementary Note 4. Hyperparameter search grid, pp. 3). Performance metrics of the tuned models reported on the test set (2021) were the RMSE, mean absolute error (MAE), and mean absolute percentage error (MAPE). Feature importance rankings were calculated for RF using the built-in *VarImp* function of the *caret* package with the robust permutation-based variable importance^48^ and for XGB using gain-based importance. Additionally, SHapley Additive exPlanations^52^ (SHAP) values were calculated and plotted using the *kernelshap* package to aid visual interpretation of the results.

### Statistical baseline models and analyses

We selected Poisson regression and boosted generalized additive models (GAM) for their established suitability in addressing count-based and nonlinear relationships in time-series data as baseline statistical models. Poisson regression was fitted with a log-link distribution and boosted generalized additive models (GAM; *mboost* package) with a negative binomial distribution (Supplementary Note 5. Generalized additive model (GAM) implementation, pp. 3) with the same set of input features and evaluated the same metrics (MAE, MAPE, RMSE) as the previously described ML models also within R (v.4.1.1). Thus, these models serve as directly comparable and interpretable benchmarks, complementing the more complex machine learning approaches in our analyses. The autocorrelation- (ACF) and partial autocorrelation function (PACF) plots of daily AIS cases were also evaluated to quantify longitudinal dependencies in the data (Supplementary Fig. 1a, b). Normally distributed variables were summarized as mean and standard deviation (SD), while non-normally distributed features were described with their median and interquartile range (IQR). Categorical variables were reported as proportions. Distributions of predictions and residuals on the test set were compared using pairwise Wilcoxon signed-rank tests with continuity correction^56^. Statistical significance was defined as two-sided *p* < 0.05, and *p*-values were adjusted for multiple testing (*N*_test_ = 10) using the Holm method (Fig. 1c). Where calculable, 95% confidence intervals (CI) were provided^49^. Figures were created using the *ggplot2* and *leaflet* R packages using color-blind safe palettes.

## Supplementary information

Supplemental materials no markups.

## Data Availability

The deidentified count data of acute ischemic stroke admissions during the seven-year study period, supporting the conclusions of this article, are available from the corresponding author upon reasonable request.

## Code availability

All details regarding the training-validation setup and hyperparameter settings are available in the companion GitHub repository of the paper (https://github.com/MIDorAI/Machine-learning-based-forecasting-of-acute-ischemic-stroke-admissions-using-weather-data).
