# Supplementary material for: Machine learning-based forecasting of daily acute ischemic stroke admissions using weather data
Source: NPJ Digit Med. 2025 Apr 25;8:225. doi: 10.1038/s41746-025-01619-w (PMC12032073; doi:10.1038/s41746-025-01619-w)
Supplement: Supplementary file 1 — Supplemental materials no markups. [file 41746_2025_1619_MOESM1_ESM.pdf]

Supplementary information

Table of contents

Supplementary notes 2
Note 1. Klima-Michael model and perceived temperature (PT) 2
Note 2. Patient matching 2
Note 3. Time-stratified nested cross-validation setup 3
Note 4. Hyperparameter search grid 3
Note 5. Generalized additive model (GAM) implementation 3
Note 6. Aggregation methodology for the heatmap in figure 3 4
Supplementary tables 5
Supplementary Table 1 5
Supplementary Table 2 6
Supplementary figures 7
Supplementary Figure 1. 8
Supplementary Figure 2. 10
Supplementary Figure 3. 11
Supplementary References 12

Supplementary notes

Note 1. Klima-Michael model and perceived temperature (PT)

Perceived temperature (PT) is an index that jointly considers factors like air temperature, humidity, wind velocity, and radiation fluxes to quantify human thermal perception.[1] PT was derived from the Klima-Michael model, which uses an energy balance approach to describe the complex interactions of meteorological components on the body's thermal equilibrium (German Weather Service (DWD), Glossary: K-Climate-Micheal model). By considering the variables relevant to the human heat balance, this model can be used to describe the temperature perception of an average person in terms of comfort, heat stress, and cold stress. PT was defined as the equivalent air temperature in an outdoor environment for a male reference subject (35 years, 1.75 m, 75 kg) with an internal heat production of 135 W/m^2 (walking at 4 km/h on flat ground) in specific conditions (50% of relative humidity) and a reduced wind velocity (slight breeze).[1] It is assumed that clothing is adapted to achieve thermal comfort. PTs between 0 and 20 °C mean comfort, <0 °C create a cold, and >20 °C a warm feeling, respectively.

DWD was among the first national weather services to operationalize a complete heat budget model specifically for human biometeorology applications (Staiger et al., Blazejczyk et al.).[1,2] Unlike UTCI and WBGT, PT is computationally efficient for high spatial and temporal resolutions and requires only minimal meteorological inputs, such as those provided by local weather stations in our area.

Comparative studies in South-West Germany (Staiger et al., Muthers et al.)[1,3] found PT to strongly correlate with UTCI (r=0.89–0.92) but with greater accessibility due to its direct link to a thermo-physiological perception scale (Blazejczyk et al.).[2] In contrast, WBGT and UTCI exhibit notable weaknesses: WBGT fails to account for dynamic conditions like wind and solar radiation, while UTCI significantly diverges at temperatures below 0°C, giving much lower values.

PT values, however, differ minimally from air temperature (−5 to +5 K) and can be calculated even in architecturally complex areas by assuming an unshaded plain. These attributes make PT season-independent, calibration-free, and ideal for multi-center applications, emphasizing its suitability for evaluating population-level weather impacts in this study (Staiger et al., Blazejczyk et al.).[1,2]

Note 2. Patient matching

Postal codes were used to link local weather patterns to the admission date and time. In case of missing or incomplete postal codes, it was replaced with that of the clinic's location (postal code). This information was then used to perform weather data extraction and geospatial matching for downstream analyses. If patients had multiple visits to the emergency department or outpatient ambulance, only the inpatient visit to the hospital was considered.

Note 3. Time-stratified nested cross-validation setup

A time-stratified 5x5-fold nested cross-validation (CV) setup with a training-validation set ranging from 2015 to 2020 and 2021 serving as the test set was employed in the study. This was implemented using the caret[4] package in the open-source R statistical programming language (v.4.1.1, R Core Team, Vienna, Austria). The root mean square error (RMSE) was used as loss function. In each fold, an additional year was incrementally added to the training set (sliding window approach). For consistency, each year was standardized to 365 days by excluding the leap year days (i.e. 2016, 2020; n=2). Weather input variables utilized in all the models underwent standardization through scaling and centering, respectively. Hyperparameters were tuned using predefined set of parameters within a grid search for the respective ML models.

Note 4. Hyperparameter search grid

The respective hyperparameters of the investigated shallow machine learning algorithms and GAM were tuned in the training-validation set within an additional nested CV as a tuning loop using either the built-in tuning option of the model function (`gamboost(…, control = boost_control(mstop = 1000L, nu = 0.01))`) or the framework (`train(…, tuneGrid = tunegrid)`) of the of the caret[4] package. In detail, the following tuning grids (`tunegrid`) and hyperparameters were used:

- random forest (RF): `expand.grid(.mtry = seq(17, 20, 1), .ntree = seq(500, 1000, 2000), .nodesize = 5)`
- support vector regression (SVR): `expand.grid(C = c(0.25, .5, 1), sigma = 0.1)`
- extreme gradient boosting (XGB):

```
expand.grid(
    nrounds = c(100, 200),
    max_depth = c(1, 3, 6, 9),
    colsample_bytree = seq(0.5,0.9, length.out = 5),
    eta = c(.2, .1, .05, .01),
    gamma = 0,
    min_child_weight = 1,
    subsample = 1
)
```

The best internally cross-validated ML-model was then used to predict the admission case counts in the test set. Further details and full R scripts can be found in the companion GitHub repositories of the paper (*https://github.com/MIDorAI/Machine-learning-based-forecasting-of-acute-ischemic-stroke-admissions-using-weather-data* and *https://github.com/medizininformatik-initiative/Projectathon6-miracum1/tree/master/step2*).

Note 5. Generalized additive model (GAM) implementation

During the training of the GAM model, the selection of specific basis functions for each variable was determined by the count of unique values present within them. When the unique values were less than 24, We used the default (reduction score) variable importance for GAM provided in the *mboost* package. Reduction score quantifies the individual contribution to risk reduction of each base-learner and can thus be used to compare the importance of different variables in the model. Higher scores [0-1] indicate a greater impact on error reduction.

Note 6. Aggregation methodology for the heatmap in figure 3

The threshold values were extracted from the inflection points of the Shapley additive explanations plot described in **Fig. 3b**. Faceted heatmap (**Fig. 3c**) was created to visualize the seasonal distribution in the training data (2015-2020). The number of days that the respective condition has occurred was calculated by jointly aggregating at yearly and weekly levels (matrix: 7 [years] x 52 [weeks] rows by 12 columns [respective conditions]). This matrix was then reduced to the weekly level to calculate the median number of days these conditions occurred across different years (aggregated over 2015-2020) within the respective 52 weeks of the aggregated year (matrix: 52 rows by 12 columns). Using this matrix, the heatmap was plotted and color-coded depending on how many days the respective weather condition occurred and whether it had a protective (negative Shapley value, blue) or harmful (positive Shapley value, red). Concurrently, the median number of AIS admissions during that respective week was compared to the quarterly median and color-coded in the delta of weekly vs. quarterly median legend (-3 to +5, blue to red).

Supplementary tables

Supplementary Table 1. **Summary table of the top six predictor estimates of the Poisson regression model.**

**Predictor variable**	**OR**	**95% CI**	**p-value**
Lag-5 mean cloud cover [%]	0.97	0.93-0.98	0.0032
Lag-1 mean pressure (P_{mean_lag1}) [hPa]	0.45	0.24-0.81	0.0076
Max. wind gust (V_{max}) [m/s]	1.02	1.00-1.04	0.017
Mean cloud cover [%]	0.96	0.94-0.99	0.018
Lag-2 min temperature (T_{min_lag2}) [°C]	1.10	1.01-1.20	0.025
Min. relative humidity [%]	1.00	1.00-1.05	0.039

95% CI: 95% confidence interval; OR: odds ratio; min.: minimum; max.: maximum.

Supplementary Table 2. **Summary table of the different features that are utilized in the fitted statistical and machine learning models.**

Type	Feature [Unit]	Abbreviation	Variable conversion
Weather	Pressure [hPa]	$P_{mean \mid min \mid max \mid \_lag1-7}$	Mean, min, max, and lagged 1-7 days
	Vapour pressure [hPa]		Mean, min, max, and lagged 1-7 days
	Temperature [°C]	$T_{mean \mid min \mid max \mid \_lag1-7}$	Mean, min, max, and lagged 1-7 days
	Dewpoint temperature [°C]		Mean, min, max, and lagged 1-7 days
	Perceived temperature [°C]	$PT_{mean \mid min \mid max}$	Mean, min, max
	Humidity [%]		Mean, min, max, and lagged 1-7 days
	Humid temperature [°C]		Mean, min, max, and lagged 1-7 days
	Sunshine duration [h]		Total and lagged 1-7 days
	Precipitation height [m]		Mean, min, max, and lagged 1-7 days
	Cloud cover [%]		Status signal
	Wind speed [m/s]	$V_{mean \mid min \mid max \mid \_lag1-7}$	Mean, min, max, and lagged 1-7 days
	Wind gust [m/s]	$V_{gust\_ \mid mean \mid min \mid max \mid \_lag1-7}$	Mean, min, max, and lagged 1-7 days
	Wind direction [°]		Mean and lagged 1-7 days
Calendar	Year		Status with values 2015 -2021
	Weekday status		Weekday or weekend (Saturday-Sunday)
	Week number		Status with values 1-52
	Holiday status		Yes/No

min.: minimum; max.: maximum; lagged 1-7 days: lag-1 to lag-7. Abbreviations of most relevant weather parameters: P: pressure; T: temperature; PT: perceived temperature; V, wind or gust speed.

Supplementary figures

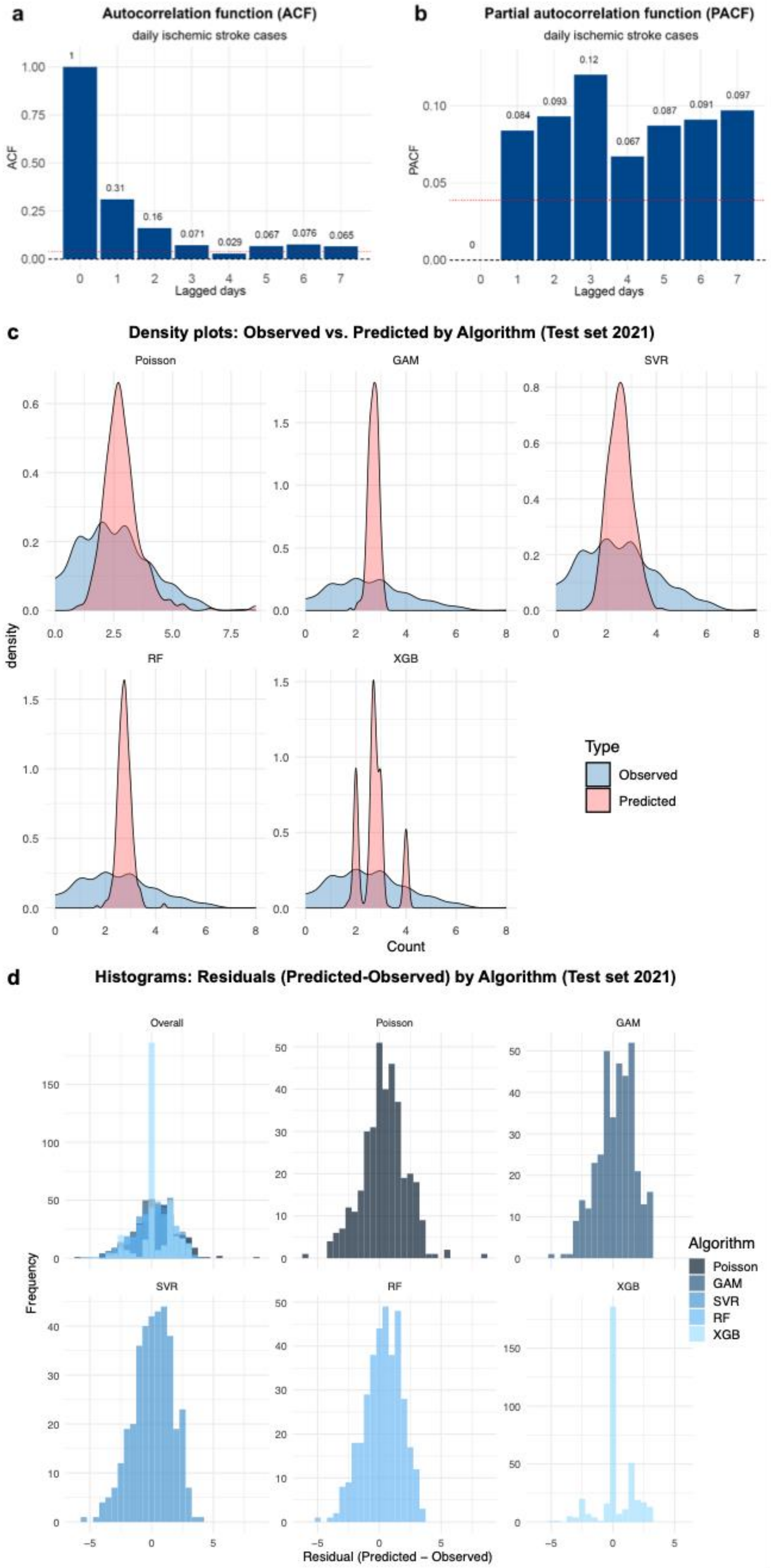

Supplementary Figure 1. **Autocorrelation- (ACF) and partial autocorrelation functions (PACF) plots, density distributions of predicted and observed and histograms of residuals of daily ischemic stroke admissions by algorithm.** The daily number of ischemic stroke admission were converted into time series, and the corresponding (**a**) ACF and (**b**) PACF with p values of the one- to seven-day lagged components were calculated and visualized as bar graphs. ACF p values were: $p_{lag-1}=0.31$, $p_{lag-2}=0.16$, $p_{lag-3}=0.071$, $p_{lag-4}=0.029$, $p_{lag-5}=0.067$, $p_{lag-6}=0.076$, and $p_{lag-7}=0.065$. PACF p values were: $p_{lag-1}=0.084$, $p_{lag-2}=0.093$, $p_{lag-3}=0.12$, $p_{lag-4}=0.065$, $p_{lag-5}=0.087$, $p_{lag-6}=0.091$, and $p_{lag-7}=0.097$. The ACF p value dropped considerably after lag-1, indicating that the influence of past number of admissions on current admissions quickly diminished after the one to two days. The PACF values did not have sharp cut-off and remained low, suggesting no strong autoregressive structure in the underlying data, making moving average or autoregressive components unnecessary for this analysis.[5] Consequently, shallow machine learning models were applied during downstream analyses. (**c**) Density plots of predicted (light red) and observed (light blue) daily AIS admissions faceted by ML algorithm. (**d**) Histograms of residuals (predicted-observed counts) overall (upper-left) and faceted by algorithm (shades of blue).

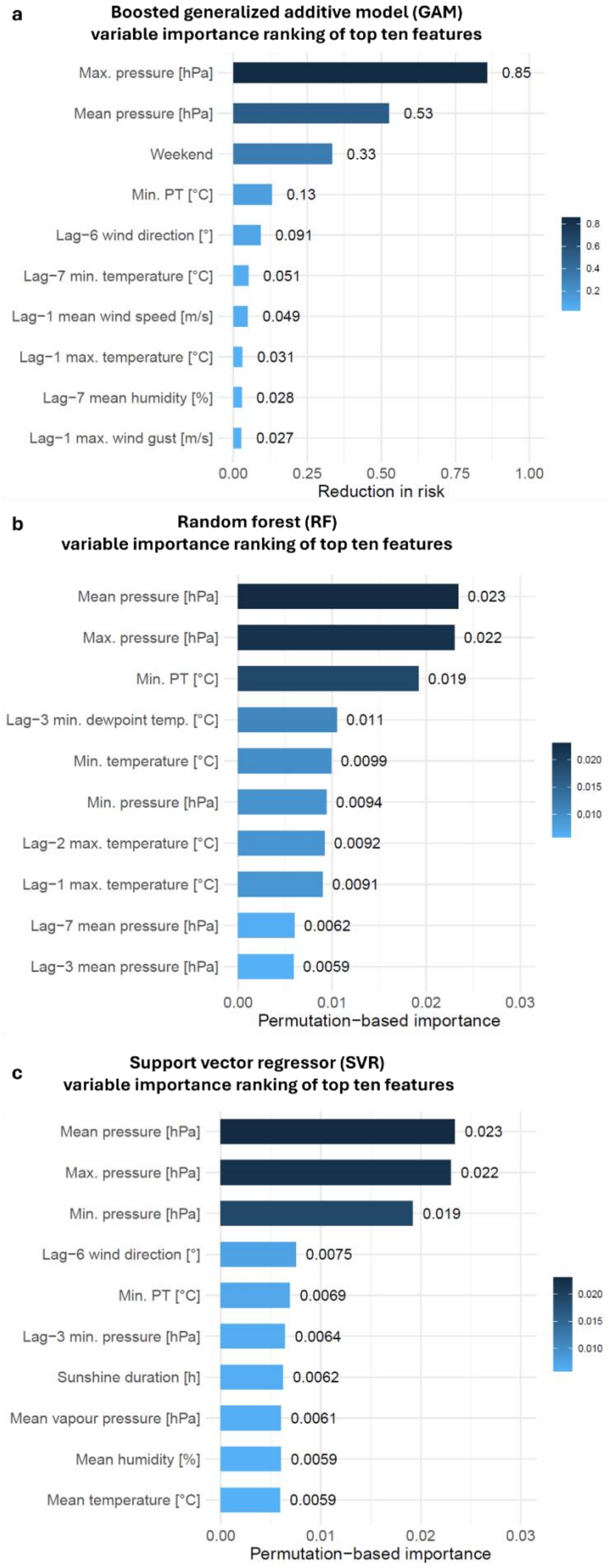
a
Boosted generalized additive model (GAM)
variable importance ranking of top ten features
Max. pressure [hPa] 0.85
Mean pressure [hPa] 0.53
Weekend 0.33
Min. PT [°C] 0.13
Lag−6 wind direction [°] 0.091
Lag−7 min. temperature [°C] 0.051
Lag−1 mean wind speed [m/s] 0.049
Lag−1 max. temperature [°C] 0.031
Lag−7 mean humidity [%] 0.028
Lag−1 max. wind gust [m/s] 0.027
0.00 0.25 0.50 0.75 1.00
Reduction in risk
0.8 0.6 0.4 0.2
b
Random forest (RF)
variable importance ranking of top ten features
Mean pressure [hPa] 0.023
Max. pressure [hPa] 0.022
Min. PT [°C] 0.019
Lag−3 min. dewpoint temp. [°C] 0.011
Min. temperature [°C] 0.0099
Min. pressure [hPa] 0.0094
Lag−2 max. temperature [°C] 0.0092
Lag−1 max. temperature [°C] 0.0091
Lag−7 mean pressure [hPa] 0.0062
Lag−3 mean pressure [hPa] 0.0059
0.00 0.01 0.02 0.03
Permutation−based importance
0.020 0.015 0.010
c
Support vector regressor (SVR)
variable importance ranking of top ten features
Mean pressure [hPa] 0.023
Max. pressure [hPa] 0.022
Min. pressure [hPa] 0.019
Lag−6 wind direction [°] 0.0075
Min. PT [°C] 0.0069
Lag−3 min. pressure [hPa] 0.0064
Sunshine duration [h] 0.0062
Mean vapour pressure [hPa] 0.0061
Mean humidity [%] 0.0059
Mean temperature [°C] 0.0059
0.00 0.01 0.02 0.03
Permutation−based importance
0.020 0.015 0.010

Supplementary Figure 2. **Variable importance of boosted generalized additive model (GAM), random forest (RF), and support vector regressor (SVR).** The bar plots indicate the top ten variables of the fitted **(a)** GAM, **(b)** RF, and **(c)** SVR models for daily acute ischemic stroke admissions, respectively. GAM identified maximum (P_{max}) and mean pressure (P_{mean}) as the top two variables, while weekend status (Saturday or Sunday) was selected as the third most important variable. GAM also included a combination of variables based on wind, temperature and humidity among the top ten variables. RF and SVR identified also mean (P_{mean}) and maximum pressure (P_{max}) as the two top variables. Similar to the extreme gradient boosting (XGB) model, RF also revealed minimum perceived temperature (PT_{min}) as the third most important variable. SVR selected minimum pressure (P_{min}) as the third most important variable. Overall, RF mainly highlighted temperature and pressure-related features in the top ten. In contrast, SVR included a combination of variables based on wind, sunshine, humidity, and temperature, similar to GAM.

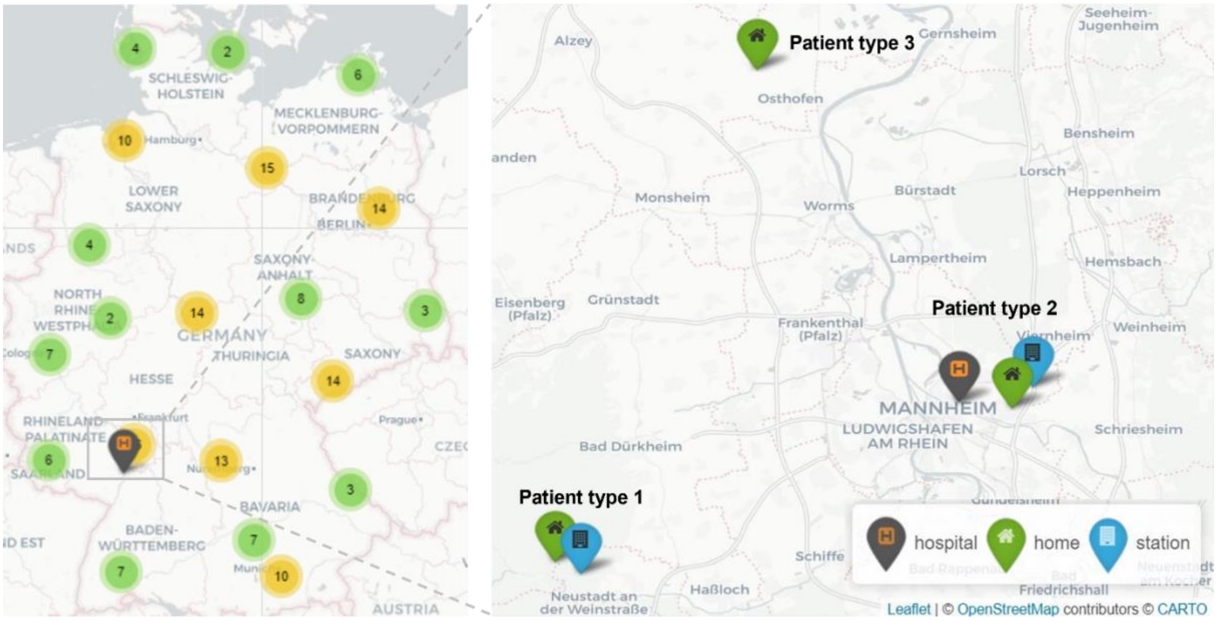

Supplementary Figure 3. **Distribution of German weather stations and patient types identified during geospatial matching.** Weather stations ($n_{station}$=135) that had full coverage of data for the seven years (2015-2021) of all selected variables (n_{var}=133) were chosen. When stations were close to each other, they were clustered together and shown as number of stations in that region in circles (number of towers <10: green; ≥10: yellow). The location of University Medical Center (UMC) was indicated with the gray pinpoint with orange H. Three different patient types were identified during the complex geospatial matching: 1) type 1 patients had a different measurement station (blue pinpoint) mapped to their home locations (green pinpoint) than the closest station to the UMC; 2) For type 2 cases, the same station was mapped as closest to both home and UMC locations (<20km away); 3) type 3 patients also matched to the same station, but their distance was >20 km to UMC and its reference station.

Supplementary References

1. Staiger, H., Laschewski, G. & Grätz, A. The perceived temperature - a versatile index for the assessment of the human thermal environment. Part A: scientific basics. *International journal of biometeorology* **56,** 165–176; 10.1007/s00484-011-0409-6 (2012).
2. Blazejczyk, K., Epstein, Y., Jendritzky, G., Staiger, H. & Tinz, B. Comparison of UTCI to selected thermal indices. *International journal of biometeorology* **56,** 515–535; 10.1007/s00484-011-0453-2 (2012).
3. Muthers, S., Laschewski, G. & Matzarakis, A. The Summers 2003 and 2015 in South-West Germany: Heat Waves and Heat-Related Mortality in the Context of Climate Change. *Atmosphere* **8,** 224; 10.3390/atmos8110224 (2017).
4. Kuhn & Max. Building Predictive Models in R Using the caret Package. *Journal of Statistical Software* **28,** 1–26; 10.18637/jss.v028.i05 (2008).
5. Box, G. E. P., Jenkins, G. M., Reinsel, G. C. & Ljung, G. M. *Time series analysis. Forecasting and control* (John Wiley & Sons, Inc, Hoboken, New Jersey, 2016).
